# Effect of the Interior Fill Percentage on the Deterioration of the Mechanical Properties of FFF-3D-Printed PLA Structures

**DOI:** 10.3390/polym17060828

**Published:** 2025-03-20

**Authors:** Akira Yamada, Kanta Tatebe

**Affiliations:** 1 Department of Mechanical Engineering, Graduate School of Engineering, Aichi Institute of Technology, Yachigusa 1247, Yakusa-cho, Toyota 470-0392, Aichi, Japan; 2 Department of Mechanical Engineering, Faculty of Engineering, Aichi Institute of Technology, Yachigusa 1247, Yakusa-cho, Toyota 470-0392, Aichi, Japan

**Keywords:** poly (lactic acid) (PLA), fused filament fabrication (FFF), immersion, water content, mechanical property, deterioration, interior fill percentage (IFP), infill, internal gap

## Abstract

Poly (lactic acid) (PLA), a biodegradable polymer, is widely used in medical applications, particularly for 3D-printed tissue engineering scaffolds. The fused filament fabrication (FFF) 3D printer is an available processing tool for PLA. The nozzle scan pattern and interior fill percentage (IFP) considerably influence the mechanical properties of formed structures and may have dominant effects on the rates at which the mechanical properties of PLA deteriorate. When the IFP is set to a low value, such as 80%, internal gaps form within the structure, leading to different deterioration patterns compared to structures formed under the IFP 100% condition. In this study, we fabricated test pieces with an FFF 3D printer using three different nozzle scan patterns. After immersing the test pieces in phosphate buffer saline (PBS) for up to 120 days, the water content was measured and the test pieces underwent tensile testing to determine the tensile strength, elastic modulus, and breaking energy. Both the deterioration rate and water uptake rate varied among the different nozzle scan patterns used for the fabrication. For the test pieces formed with internal gaps, the water uptake and deterioration proceeded in two stages. The deterioration rate of the structures with internal gaps was faster than that of the fully filled structures. The data obtained in this study will be useful for the design of PLA structures applied in tissue engineering.

## 1. Introduction

Poly (lactic acid) (PLA) is a biodegradable plastic synthesized from lactide, a compound extracted from plants such as corn and cassava. Since the establishment of large-scale PLA synthesis technology [1,2], PLA has been successfully applied in bone fixation devices for mending fractures, as well as wide-ranging tissue engineering scaffolds for the treatment of deficits of the bone and other tissues [3,4,5,6,7,8]. The mechanical deterioration of the material is an important parameter to consider in load-bearing structures, such as bone. Numerous attempts have been made to incorporate 3D printing technologies into the methods used to process PLA for the aforesaid applications [9,10,11,12,13,14,15,16,17,18].

The 3D printer is now capable of forming three-dimensional objects from the design data output from a computer-aided design (CAD) system [19]. The fused filament fabrication (FFF) 3D printer, one of the most widely used 3D printing devices in recent years, can become a powerful tool for tissue engineering applications [20]. The FFF 3D printer uses a continuous filament of a thermoplastic resin. The physical properties of PLA, such as its glass transition temperature and melting temperature, are well suited to FFF 3D printing. The melted polymer is extruded from a tiny nozzle, which is controlled by a PC in response to model data. The nozzle scan pattern is selected based on the applied forces and the desired structural density. The internal density is set by adjusting a parameter called the interior fill percentage (IFP), also termed the infill ratio, infill percentage, or infill density. In most instances, a low IFP is selected to save material and reduce the fabrication time. A structure formed by FFF 3D printing has internal gaps between the adjacent extruded filaments. These gaps widen in a PLA structure fabricated using a lower IFP, which both reduces the structural strength and influences the moisture behavior when the structure is immersed.

The decomposition of PLA occurs through hydrolysis, leading to a corresponding decline in the mechanical properties [3,4]. The deterioration rate of PLA structures depends on a range of factors, such as the temperature, composition, and pH of the immersion solution, as well as the size of the immersed structure [21,22,23]. The presence of internal gaps of the above type can accelerate structural deterioration by enhancing moisture absorption and diffusion through the PLA matrix.

The mechanical properties of FFF-3D-printed PLA structures have been evaluated by tensile tests [24,25,26,27,28,29,30] and flexural tests [31]. The strength of an FFF-3D-printed structure varies in accordance with the nozzle scan pattern [29,32,33,34,35,36]. We previously reported that the tensile strength of an FFF-3D-printed structure printed in parallel to the longitudinal direction at IFP 100% was nearly identical to a structure formed by the injection molding (IM) method [37]. The deterioration rate of mechanical properties of saline-immersed structures varied with the nozzle scan pattern and IFP [37,38]. The deterioration rate can be expected to vary in structures formed with lower IFPs (such as 80%), as these structures have gaps between the scanned lines, allowing the surrounding moisture to easily permeate and diffuse into the interior of the material. While these relationships have yet to be fully understood, the deterioration rate of a PLA structure with internal gaps is an important parameter to consider in designing the structure. Unlike previous studies [37,38], this work specifically examines how moisture behaves in PLA structures with various internal structures. We created test pieces with unique internal gap configurations by employing different nozzle scan patterns and reduced IFPs during 3D printing. Understanding the relationship between the moisture behavior and mechanical property degradation during immersion will advance PLA applications in new directions. Another limitation of our previous study was the immersion period of the structures tested [37,38], as no tests were performed on structures immersed for longer than 90 days. Tensile testing could not be performed on severely deteriorated structures, as the test pieces broke during clamping.

As the behavior of water in the structure plays an essential role in the decomposition of PLA, we have focused on the water content and its effect on structural deterioration. PLA is a biodegradable polymer with a slow degradation rate, hence the material decomposes by bulk erosion [5,39,40,41]. The structure absorbs water into spaces created by eluted oligomers as the polymer degrades, causing a corresponding decline in the structural strength of the PLA. The water content of a PLA structure is a critical determinant of the deterioration rate, as the decomposition proceeds by hydrolysis. Studies have investigated the moisture absorption and its effects on the mechanical behavior of 3D-printed PLA structures through flexural testing of test pieces immersed into deionized water [42]. Another study examined the water uptake and tensile properties of PLA test pieces formed by the IM method, with modifications to the temperature, immersion solution, and other test conditions [43]. Some papers have reported that the water content of immersed native PLA reaches saturation at about 1% [44,45]. Overall, however, the mechanisms of the water content changes in PLA structures remain poorly understood. More particularly, the relation between these changes and the deterioration of PLA remains to be clarified in 3D-printed PLA structures.

In this study, we investigated how different nozzle scan patterns affect the mechanical deterioration rates of FFF-3D-printed PLA structures containing internal gaps. The test pieces were immersed in phosphate-buffered saline (PBS) for varying durations before undergoing water content measurements and tensile testing. We derived the mechanical properties from the stress–strain curves and conducted detailed analyses on the relationship between the water content and tensile strength. The mechanical deterioration rates varied among the different internal structures formed by the various nozzle scan patterns and IFPs, with lower IFP structures deteriorating more rapidly. A two-stage deterioration process corresponding to moisture absorption also appeared, but only in the low IFP structures containing internal gaps. Our findings provide valuable insight into the design of objects fabricated from PLA.

## 2. Materials and Methods

### 2.1. Preparation of the Test Pieces

The test piece was designed in the form of a flat dumbbell with a square cross-section measuring 3 × 3 mm, as reported previously [37]. The design was modified from the ASTM D638 Type V standard to accommodate immersion testing requirements. A digital model of the test piece was created using a three-dimensional computer-aided design (3D-CAD) system (AutoCAD, Autodesk Inc., Mill Valley, CA, USA), and the data were output in a standard triangulated language (STL) format. The test pieces were printed by an FFF 3D printer (Lepton2, MagnaRecta Inc., Tokyo, Japan) with printer control software (Simplify3D Ver. 4.0.0, Simplify3D LLC, Cincinnati, OH, USA). The printed material was a white commercially available PLA (KYORAKU Co., Ltd., Tokyo, Japan) with a filament diameter of 1.75 mm. The printer was configured with a 0.4 mm nozzle diameter, 0.2 mm layer thickness, and nozzle scan speed of 480 mm/min. The nozzle and modeling stage temperatures were set at 200 °C and 70 °C, respectively, with IFPs of either 80% or 100%.

**Figure 1:**
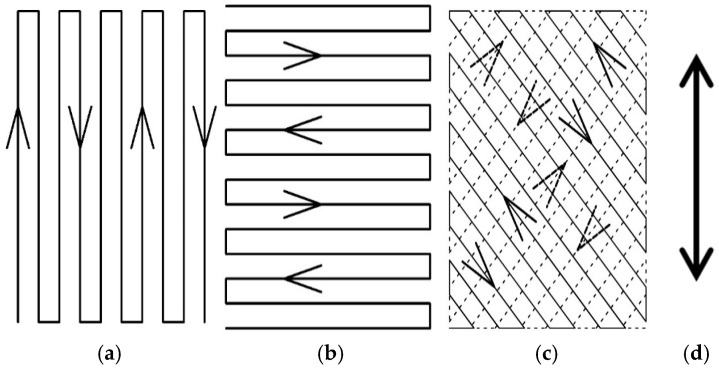
Nozzle scan patterns (**a**) P., (**b**) V., and (**c**) C. (**d**) The two-headed arrow indicates the tensile direction [37].

Figure 1 shows the three nozzle scan patterns, namely, parallel (P.), vertical (V.), and cross-hatched at opposing diagonal angles (C.) against the tensile direction [37]. We defined the test piece formed under the conditions of P. and IFP 80% as P._80%_, and applied V._80%_, C._80%_, P._100%_, and V._100%_, correspondingly.

After the 3D printing, each test piece to be analyzed by the immersion testing was immersed in a small polystyrene case containing 8 mL of PBS (0.1 mol/L, pH 7.0, 168-27155, FUJIFILM Wako, Tokyo, Japan). All of the immersed test pieces (P._80%_, V._80%_, C._80%_, P._100%_, and V._100%_) were incubated at 37 °C for 14, 30, 45, 75, or 90 days, and additional P._80%_, V._80%_, and C._80%_ test pieces were immersed for 120 days.

### 2.2. Measurement of the Moisture Content

The moisture content of the test pieces was evaluated by a heat drying moisture analyzer using a halogen heater (MS-70, A&D Company, Limited, Tokyo, Japan). The moisture analyzer was interfaced with Windows communication tools (WinCT-Moisture, Ver. 2.41) running on a PC. The moisture content was calculated using the following equation:

∆*m* = (*m*_0_ − *m*)/*m*_0_ × 100 (%), (1)

where *m*_0_ and *m* are the mass before and after drying, respectively.

The test pieces were taken out from the immersed solution and wiped with Kimwipes to remove any moisture adhering to the surfaces. Five test pieces were set on the sample pan of the moisture analyzer. The heating cycle was set for a 180 min duration using ramp drying. The temperature was gradually increased to 120 °C from room temperature (RT) (23–25 °C) over 90 min and kept at 120 °C for an additional 90 min.

### 2.3. Evaluation of Mechanical Properties

**Figure 2:**
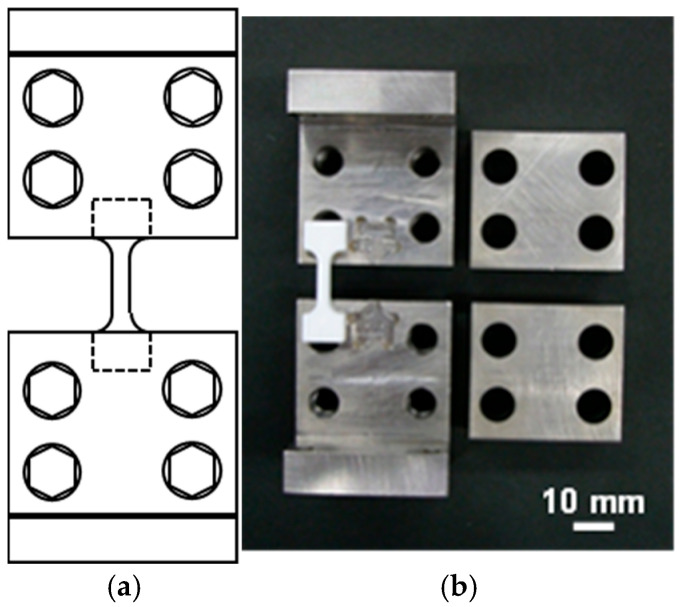
(**a**) Form of the jigs and (**b**) photograph of a jig.

The mechanical properties of the structures were evaluated by tensile testing using a universal testing machine (TENSILON RTC-1250A, ORIENTEC Co., Ltd., Tokyo, Japan). The load was measured by a 5 kN load cell (UR-5KN-D, ORIENTEC Co., Ltd., Tokyo, Japan). A diagram Figure 2a and photograph Figure 2b of the tensile test jig are shown in Figure 2. The jigs had custom-fitted indentations matching the test piece shape at the point of attachment to prevent the breakage of weakened test pieces after extended immersion.

The strain rate was 1.2 min^−1^ based on the crosshead velocity of 1.2 mm/min and gauge length of 10 mm. The preload was set at 15 N for the test pieces immersed for 14, 30, 45, and 60 days. A lower preload of 1.5 N was set for the test pieces immersed for 75, 90, and 120 days, as some test pieces immersed for 75 days broke before reaching the 15 N preload level. Load and displacement data were collected to calculate stress and strain. The dimensions of the test piece were measured before the tensile testing to calculate stress. The tensile tests were performed at RT and atmospheric pressure after the completion of moisture content tests on the same day.

The mechanical properties were evaluated using the following formulas:

(2) $$\sigma =\frac{P}{A},$$

(3) A=w×t,

(4) $$E=\frac{\sigma }{\varepsilon },$$

(5) $$\varepsilon =\frac{\Delta l}{l_{0}},$$

(6) $$U=\int_{0}^{\varepsilon b}\sigma (\varepsilon )d\varepsilon$$

where *σ* is the tensile stress (MPa), *P* is the test load (N), *A* is the outer cross-sectional area of the test piece (mm^2^), *w* and *t* are the width (mm) and thickness (mm) of the test piece, *E* is the Young’s modulus (GPa), *ε* is the strain in the tensile test (-), ∆*l* is the displacement of the cross head (mm), *l*_0_ is the gauge length (mm), *U* is the breaking energy (MJ/m^3^), and *ε_b_* is the breaking strain.

The maximum tensile stress was the highest point on the stress–strain curve, and the stress was considered to be the strength of the test piece. The Young’s modulus was calculated from the steepest slope of the tangential line of the elastic region on the stress–strain curve. The breaking energy was estimated from the area of the stress–strain curve.

## 3. Results

### 3.1. Mechanical Properties

**Figure 3:**
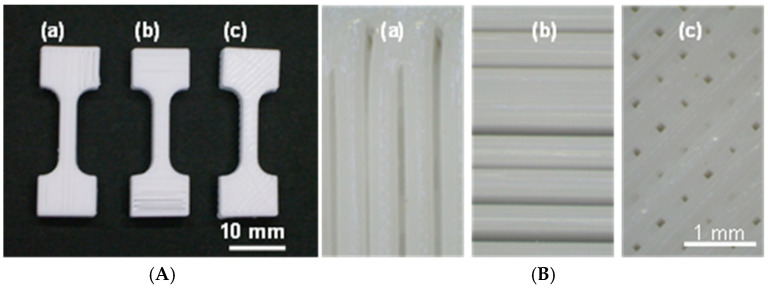
(**A**) Photograph of the (**a**) P._80%_, (**b**) V._80%_, and (**c**) C._80%_ test pieces. (**B**) Magnified images of the top surfaces of the aforesaid.

Figure 3A shows photographs of the three IFP 80% test piece types, P._80%_, V._80%_, and C._80%_. The gaps between the neighboring filaments are clearly visible. Figure 3B shows magnified images of typical sections of the top surfaces using optical micrographs of the three test piece types. We can clearly find traces of nozzle scanning for each pattern, as well as gaps or holes on the surface of the test pieces fabricated at the IFP 80% setting.

**Figure 4:**
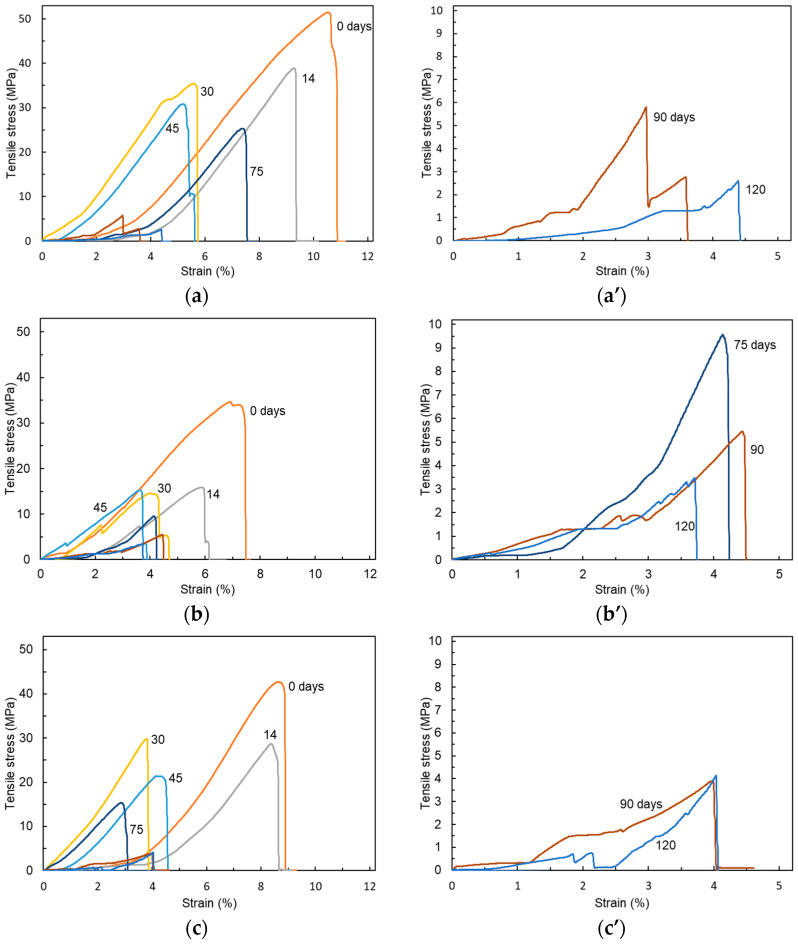
Stress–strain curves of the (**a**) P._80%_, (**b**) V._80%_, and (**c**) C._80%_ test pieces over 0–120 days of immersion. The curves plotting maximum tensile stress of less than 10 MPa are enlarged in (**a’**–**c’**).

Figure 4 shows representative changes in the stress–strain curves induced by immersion in the tensile tests of the Figure 3a P._80%_, Figure 3b V._80%_, and Figure 3c C._80%_ test pieces. The strain increased the stress up to the point of sudden breakage in every type of test piece. The maximum stress dropped as the immersion period increased in every type of test piece.

**Figure 5:**
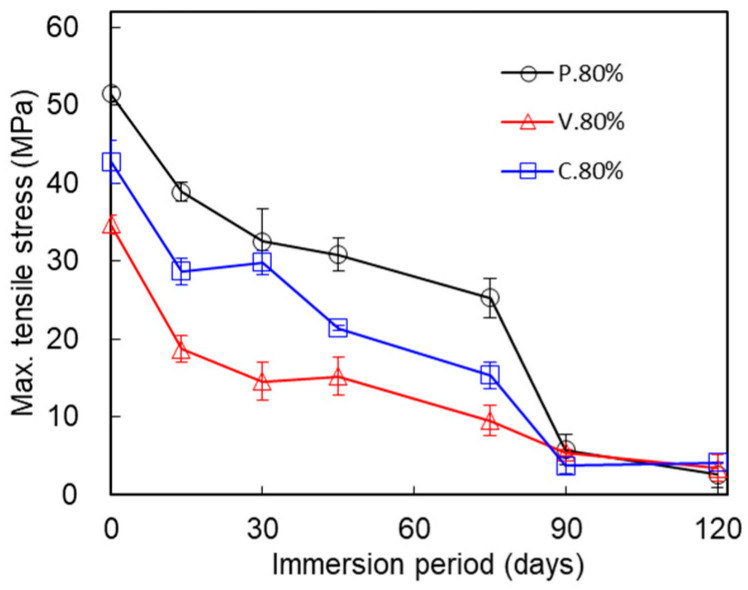
Comparison of the changes in the maximum tensile stresses of the P._80%_, V._80%_, and C._80%_ test pieces during the immersion. Average ± S.D., *N* = 5.

Figure 5 compares the changes in the maximum tensile stresses for the three test piece types printed at the IFP 80% setting. The maximum tensile stress of non-immersed test pieces (0 days) varied according to the test piece type: P._80%_, 51.45 ± 0.88; V._80%_, 34.67 ± 2.76; and C._80%_, 42.73 ± 1.22 (MPa). The maximum tensile stresses after immersion for 14, 30, 45, 75, 90, and 120 days were as follows: (14 days) P._80%_, 38.87 ± 1.20; V._80%_, 18.74 ± 1.69; C._80%_, 28.71 ± 1.66 (MPa); (30 days) P._80%_, 32.52 ± 4.23; V._80%_, 14.55 ± 1.55; C._80%_, 29.83 ± 2.46 (MPa); (45 days) P._80%_, 30.85 ± 2.08; V._80%_, 15.25 ± 0.33; C._80%_, 21.43 ± 2.49 (MPa); (75 days) P._80%_, 25.32 ± 2.51; V._80%_, 9.57 ± 1.68; C._80%_, 15.35 ± 2.02 (MPa); (90 days) P._80%_, 5.81 ± 2.00; V._80%_, 5.46 ± 1.07; C._80%_, 3.75 ± 0.40 (MPa); and (120 days) P._80%_, 2.61 ± 1.57; V._80%_, 3.48 ± 0.11; and C._80%_, 4.14 ± 1.74 (MPa). The maximum tensile stress dropped steeply over immersion periods of 0–30 days and 75–90 days, and gradually over immersion periods of 45–75 days. The maximum tensile stresses remained almost equal between 90 and 120 days of immersion.

**Figure 6:**
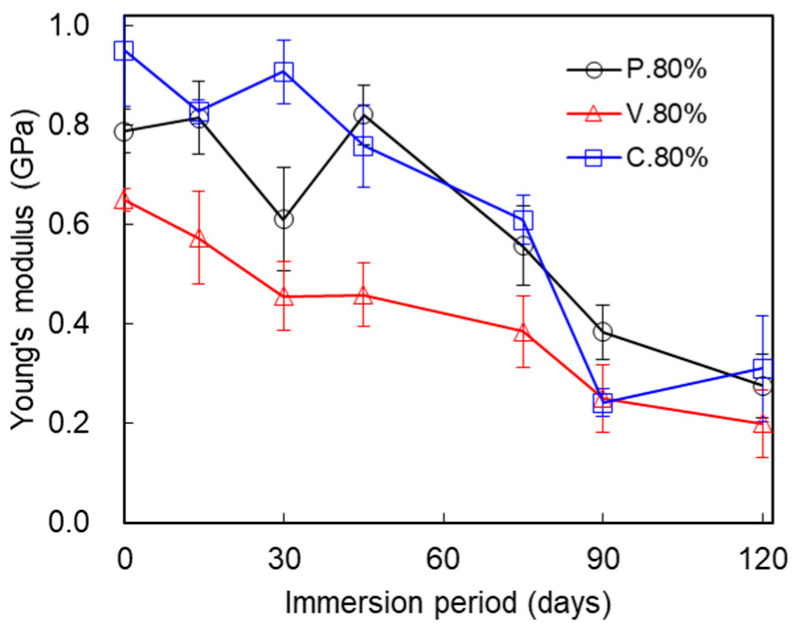
Comparison of the changes in the tensile elastic moduli of the P._80%_, V._80%_, and C._80%_ test pieces during the immersion. Average ± S.D., *N* = 5.

Figure 6 compares changes in the Young’s moduli for the three test piece types. The Young’s moduli after immersion for 0, 14, 30, 45, 75, 90, and 120 days were as follows: (0 days) P._80%_, 0.787 ± 0.045; V._80%_, 0.650 ± 0.022; C._80%_, 0.949 ± 0.112 (GPa); (14 days) P._80%_, 0.814 ± 0.073; V._80%_, 0.573 ± 0.093; C._80%_, 0.827 ± 0.024 (GPa); (30 days) P._80%_, 0.610 ± 0.104, V._80%_, 0.455 ± 0.069; C._80%_, 0.906 ± 0.065 (GPa); (45 days) P._80%_, 0.820 ± 0.061; V._80%_, 0.458 ± 0.064; C._80%_, 0.758 ± 0.082 (GPa); (75 days) P._80%_, 0.557 ± 0.081; V._80%_, 0.386 ± 0.072; C._80%_, 0.609 ± 0.048 (GPa); (90 days) P._80%_, 0.384 ± 0.055; V._80%_, 0.250 ± 0.068; C._80%_, 0.242 ± 0.028 (GPa); and (120 days) P._80%_, 0.275 ± 0.064; V._80%_, 0.199 ± 0.067; and C._80%_, 0.311 ± 0.106 (GPa). The Young’s moduli gradually dropped as the immersion period increased for all the test piece types. Though unexpected fluctuations over the immersion period were seen in the P._80%_ test pieces, the Young’s moduli dropped as a general trend.

**Figure 7:**
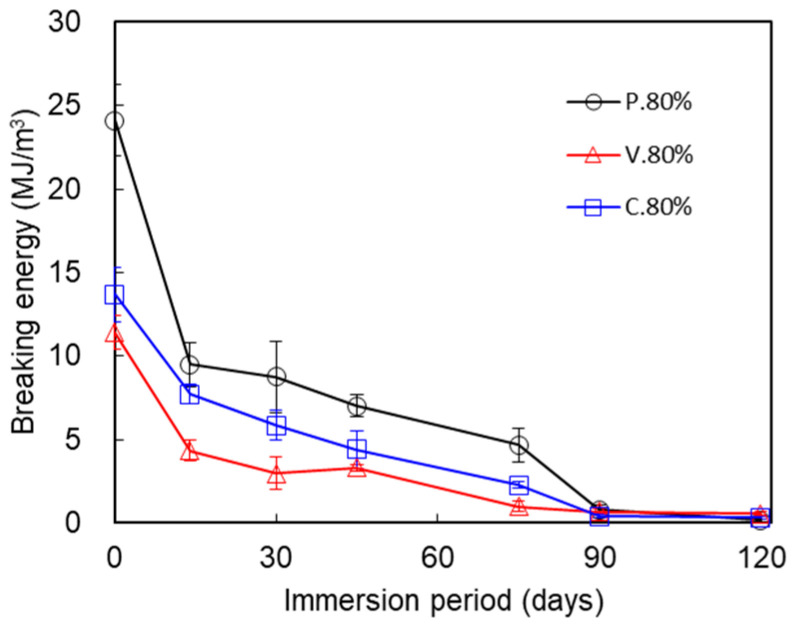
Comparison of the changes in the tensile breaking energies of the P._80%_, V._80%_, and C._80%_ test pieces during the immersion. Average ± S.D., *N* = 5.

Figure 7 compares changes in the breaking energies for the three test piece types. The breaking energies after immersion for 0, 14, 30, 45, 75, 90, and 120 days were as follows: (0 days) P._80%_, 24.1 ± 2.13; V._80%_, 11.4 ± 1.02; C._80%_, 13.7 ± 1.63 (MJ/m^3^); (14 days) P._80%_, 9.50 ± 1.30; V._80%_, 4.34 ± 0.62; C._80%_, 7.71 ± 0.57 (MJ/m^3^); (30 days) P._80%_, 8.70 ± 2.12; V._80%_, 2.97 ± 0.97; C._80%_, 5.85 ± 0.91 (MJ/m^3^); (45 days) P._80%_, 7.00 ± 0.65; V._80%_, 3.31 ± 0.19; C._80%_, 4.40 ± 1.12 (MJ/m^3^); (75 days) P._80%_, 4.70 ± 1.01; V._80%_, 0.99 ± 0.32; C._80%_, 2.28 ± 0.20 (MJ/m^3^); (90 days) P._80%_, 0.80 ± 0.27; V._80%_, 0.69 ± 0.16; C._80%_, 0.42 ± 0.12 (MJ/m^3^); and (120 days) P._80%_, 0.20 ± 0.17; V._80%_, 0.56 ± 0.11; and C._80%_, 0.31 ± 0.10 (MJ/m^3^). The breaking energies after 0 days of immersion were largest in the P. test piece. The breaking energy dropped suddenly over the immersion periods of 0–14 days and gradually over the immersion periods of 14–90 days for all three test piece types. The breaking energies for all the immersion periods were largest in the P._80%_ test pieces, then C._80%_ and V._80%_, consecutively. The breaking energies declined slightly between 90 and 120 days of immersion.

**Figure 8:**
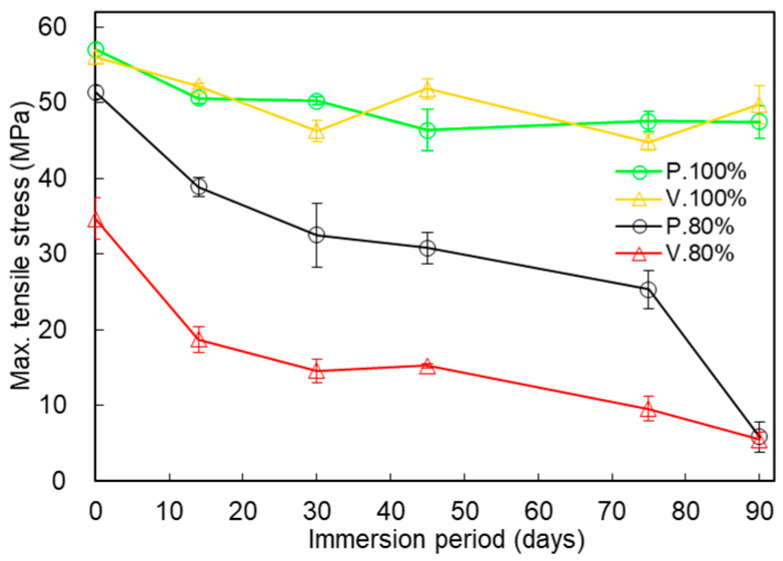
Comparison of the changes in the maximum tensile stresses of the P._100%_, V._100%_, P._80%_, and V._80%_ test pieces during the immersion. Average ± S.D., *N* = 5.

Figure 8 compares the changes in the maximum tensile stresses for the P., V., IFP 100%, and IFP 80% test piece types over 90 days of immersion. The maximum tensile stresses of the P._100%_ and V._100%_ types after 0 days were 57.04 ± 0.89 and 56.09 ± 0.84 (MPa), respectively. The maximum tensile stresses after immersion for 14, 30, 45, 75, and 90 days were as follows: (14 days) P._100%_, 50.60 ± 0.67; V._100%_, 52.24 ± 0.38 (MPa); (30 days) P._100%_, 50.26 ± 0.56; V._100%_, 46.34 ± 1.40 (MPa); (45 days) P._100%_, 46.39 ± 2.76; V._100%_, 51.87 ± 1.35 (MPa); (75 days) P._100%_, 47.55 ± 1.32; V._100%_, 44.80 ± 1.17 (MPa); and (90 days) P._100%_, 47.49 ± 2.16; and V._100%_, 49.77 ± 2.52 (MPa). For both scan patterns, the immersion brought about steep decreases in the maximum tensile stresses in the IFP 80% test pieces and gradual decreases in the IFP 100% test pieces.

### 3.2. Moisture Content

**Figure 9:**
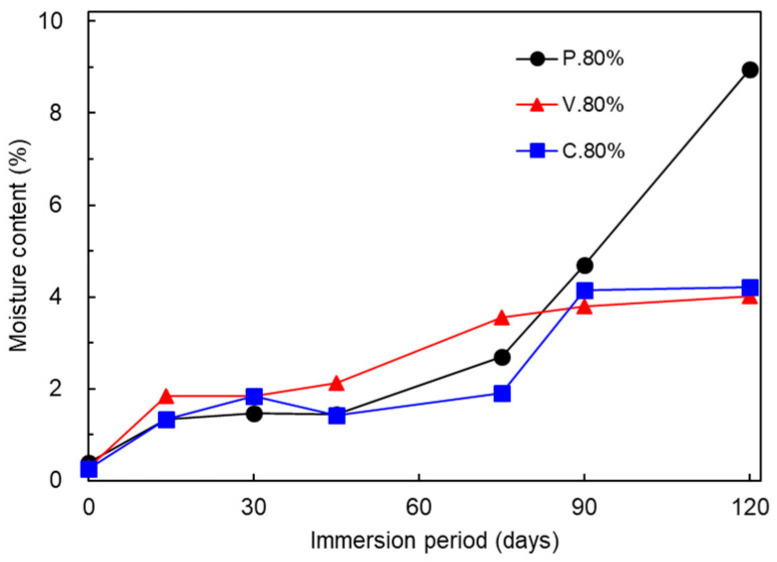
Comparison of the changes in the moisture content of the P._80%_, V._80%_, and C._80%_ test pieces.

Figure 9 compares changes in the moisture content for the P._80%_, V._80%_, and C._80%_ test pieces. The moisture contents after immersion for 0, 14, 30, 45, 75, and 90 days were as follows: (0 days) P._80%_, 0.386%; V._80%_, 0.281%; C._80%_, 0.266%; (14 days) P._80%_, 1.338%; V._80%_, 1.840%; C._80%_, 1.322%; (30 days) P._80%_, 1.466%; V._80%_, 1.835%; C._80%_, 1.828%; (45 days) P._80%_, 1.450%; V._80%_, 2.117%; C._80%_, 1.413%; (75 days) P._80%_, 2.697%; V._80%_, 3.545%; C._80%_, 1.914%; (90 days) P._80%_, 4.687%; V._80%_, 3.795%; C._80%_, 4.136%; and (120 days) P._80%_, 8.964%; V._80%_, 4.013%; and C._80%_, 4.203%. For all three test piece types, the moisture content rose to 1.32–1.84% over the 0–14 day immersion period, remained almost unchanged over the 14–45 day period, and rose gradually to 3.80–4.69% over the 45–90 day period. While the moisture content of the P._80%_ test piece rose sharply to 8.96% over the succeeding immersion up to 120 days, that of the V._80%_ and C._80%_ test pieces rose only slightly.

### 3.3. Relationships Between the Moisture Content and Mechanical Properties

**Figure 10:**
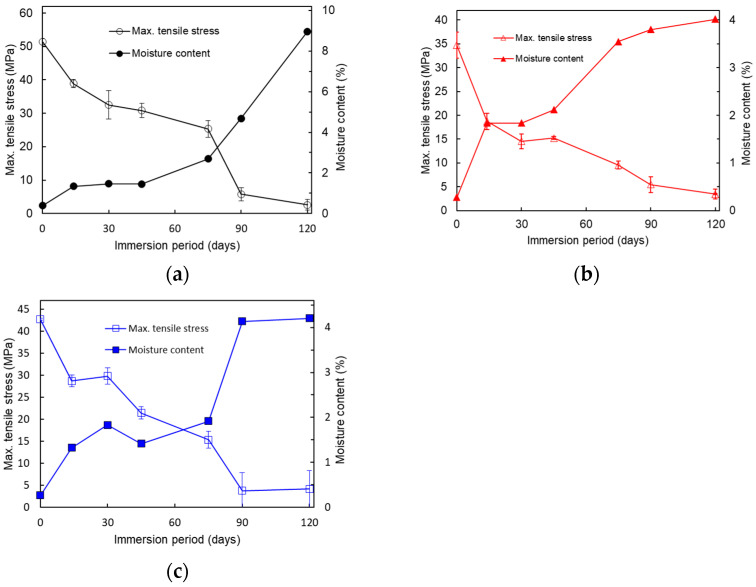
Relationships between the changes in the maximum tensile stress and moisture content of the **(a**) P._80%_, (**b**) V._80%_, and (**c**) C._80%_ test pieces during the immersion.

Figure 10 compares the relationships between the maximum tensile stress and moisture content for the Figure 10a P._80%_, Figure 10b V._80%_, and Figure 10c C._80%_ test pieces. The maximum tensile stress decreased as the moisture content increased during the immersion for all the test piece types. Both the moisture uptake and tensile strength showed two distinct stages of rapid change. For all test piece types, the first stage occurred during the initial 0–14 days of immersion. The second stage began after 75 days for the P. and C. patterns, while it started earlier, after 45 days, for the V. pattern.

## 4. Discussion

### 4.1. Deterioration of the Mechanical Properties of Structures with Internal Gaps

The mechanical properties of the IFP 80% test pieces differed among the three test piece types. At time 0 (non-immersion), the maximum tensile stress of the P._80%_ test piece was over 90.2% that of the P._100%_ test piece, while the maximum tensile stress of the V._80%_ test piece was 61.8% that of the V._100%_ test piece (Figure 8). And, as plotted in Figure 5, the maximum tensile strength was highest in P._80%_ among the three test piece types. Figure 5 clearly demonstrates that the mechanical deterioration proceeded in two stages in the three test piece types (described later). We see from Figure 6, meanwhile, that the elastic modulus of V._80%_ was the smallest among the three test piece types. The immersion tended to decrease the elastic moduli, which suggests that it gradually degraded the tensile stiffness of the structure. The breaking energies were largest in P._80%_ for each immersion period and decreased in the order of C._80%_, V._80%_ (Figure 7). The rate of decline of the breaking energy between 30 and 75 days was almost equal among the three test piece types. This finding indicates that the rate at which the breaking energy declined was unrelated to the nozzle scan pattern set for the 3D printing, at least under the IFP 80% condition. The tensile strengths of non-immersed (0 days) test pieces were largest in test piece type P._80%_, followed by C._80%_ and V._80%_ (Figure 5). The internal structure of P._80%_ consisted of filaments aligned parallel to the tensile direction, with all filaments contributing to the resistance of tensile loading. The V._80%_ was weaker than the other two types: its strength along the tensile direction was maintained only by line adhesion, and the adhesion points were prone to failure under tensile force. The strength of C._80%_ decreased with the reduction in IFP because the 45° orientation of filaments relative to the tensile direction reduced the load-bearing capacity. This hierarchical ordering of the tensile strength persisted throughout the deterioration process during immersion for up to 75 days. This pattern could be attributed to the thinner filaments composing the IFP 80% structure (to be described later). The nozzle scan pattern relative to the tensile direction appears to be a critical parameter to consider when designing structures. When the structure must withstand forces from multiple directions, designers should choose the nozzle scan pattern C.

Irregular values in the following test pieces could be observed in Figure 5, Figure 6, Figure 7, Figure 8, Figure 9 and Figure 10: in V._80%_ at 30 days and C._80%_ at 45 days in Figure 5; in P._80%_ at 14, 30, and 45 days, V._80%_ at 30 and 45 days, and C._80%_ at 14, 30, 90, and 120 days in Figure 6; in V._80%_ at 45 days in Figure 7; in P._100%_ at 45 days and in V._100%_ at 30 and 45 days in Figure 8; and in V._80%_ at 30 days and in C._80%_ at 30, 45, and 90 days in Figure 9. Some of these irregularities fell within the error margins. While the initial increase in Young’s modulus for P._80%_ between 0–14 days may have resulted from material tightening due to water absorption (Figure 6), several other factors might also explain these irregularities. Dimensional variations in the test pieces during 3D printing may have affected tensile strength (Figure 5 and Figure 8). The Young’s modulus may have been easily influenced by the test piece clamping condition, such as slippage or contact area damage. Conditions of this type were clearly observable in the IFP 80% test pieces immersed for the longer periods. Minor fluctuations in incubation temperature may have affected the rate of deterioration. The variations in the breaking energy corresponded to the differences in tensile elongation observed at failure (Figure 7). The water content measurements were susceptible to residual moisture in the structural gaps or on the outer surfaces. These irregular results may also have stemmed from ambient humidity during test piece preparation, as well as additional factors such as the self-catalytic action of the degraded oligomers [46].

Unique relations among the changes in mechanical properties can be seen in Figure 5, Figure 6 and Figure 7. While the maximum tensile strengths and breaking energies decreased substantially for all three IFP 80% test pieces in the initial 14 days, the Young’s moduli showed no significant decline. While the strength and toughness deteriorated rapidly in the early stages of immersion, stiffness degraded at a much slower rate. These findings provide valuable insights for the establishment of appropriate safety factors in the design of PLA objects. The mechanisms responsible for the slower decline in Young’s modulus, however, remain unclear at present.

### 4.2. Moisture Behavior in a PLA Structure Formed by 3D Printing

**Figure 11:**
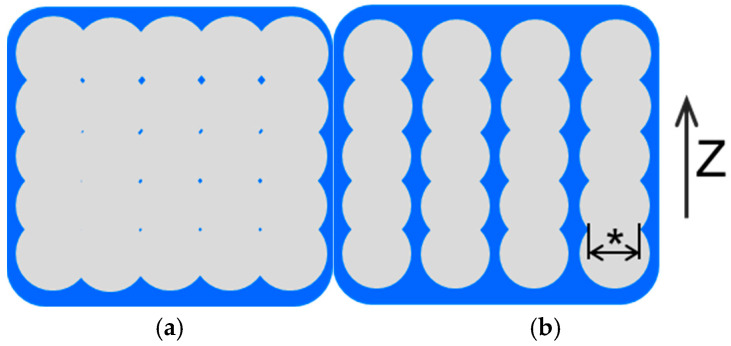
Schematic diagrams of the cross-sectional model for (**a**) P._100%_ and (**b**) P._80%_. The asterisk (*) indicates the thickness at the lowest point.

Moisture behavior is a concern in a PLA structure, as the PLA deteriorates through the process of hydrolysis. The moisture is thought to permeate from the surface of the structure and diffuse through the polymer material. The internal structures and available surface area are formed by the nozzle scan pattern. A lower IFP leaves gaps between the scanned lines and increases the available surface area of the structure as a result. The moisture content of the P._80%_, V._80%_, and C._80%_ test pieces ranged from 3.80% to 4.69% after 90 days of immersion (Figure 9). This range was clearly larger than that of the IFP 100% test pieces measured by a similar method (1.6–1.7%) [37]. These results indicate that the test pieces formed under the IFP 80% condition absorb more moisture than the IFP 100% test pieces. As shown in Figure 11, the IFP 80% structure had double the contact area with moisture than the IFP 100% structure. The thinner structure hastens water uptake and permeation into the material, thereby accelerating the structural deterioration. The moisture content of the P._80%_ test piece continuously rose after 90 days of immersion, whereas that of the V._80%_ and C._80%_ test pieces stayed flat (Figure 9). To explain this, we surmise that the test pieces formed by the P._80%_ scan pattern had a larger contact area with moisture than the V. and C. test pieces formed under the IFP 80% condition, which accelerated the degradation of the material. We also surmise that the capillary phenomenon formed between the filaments accelerated the deterioration of the mechanical properties.

Hydrolysis is one of the main mechanisms of PLA degradation, along with thermal decomposition and photodegradation. Hydrolysis cleaves the ester groups of the main chain, which reduces the molecular weight (MW) and brings about corresponding declines in the mechanical strength. We know from previous studies that the induction period preceding significant hydrolytic degradation is longest for weight loss, shorter for degradation of mechanical properties, and shortest for MW loss [47,48]. In our study, meanwhile, the increase in the water content could serve as a proxy for the weight change. A mechanism distinctly different from that observed in the previous research appears to be at work, as no induction period could be observed between the tensile strength reduction and moisture content increase for any of the test piece types (see Figure 10). This difference can probably be explained by the internal gaps, as they reduced the thickness of the structures to approximately 0.4 mm and correspondingly accelerated the diffusion of moisture to the centers.

We set the nozzle diameter to 0.4 mm and the layer thickness to 0.2 mm in the 3D printer settings to form the test pieces. The typical line widths in P._80%_ measured by microscopic observation were 0.38–0.4 mm, excluding irregular side-by-side line adhesion and thinner lines caused by the unstable extrusion of the melted polymer (Figure 3). The thickness at the lowest points was estimated to be around 0.32–0.36 mm, adjusting for a surface roughness of 0.2–0.3 mm on each side (Figure 11). Water immersion to the material center was therefore substantially faster in the P._80%_ structures than in the P._100%_ structures, as the latter had a thickness of 3 mm. The thinnest points were likely to serve as action sites where the structural deterioration was initiated. We therefore surmise that the layer adhesion failures occurred more readily in the P._80%_ structures than in the P._100%_ structures, accelerating the mechanical deterioration in the former. In spite of the different internal structures in V._80%_ and C._80%_, the deterioration appeared to proceed by similar mechanisms in the two cases.

### 4.3. Relationships Between the Moisture Content and Rate of Mechanical Property Deterioration

Our analysis showed that the deterioration proceeded in two stages across all three test piece types (Figure 5 and Figure 10). The timing of the second stage, characterized by moisture uptake and a falloff in tensile strength, varied among the test piece types. This timing variation suggests that the onset of the second stage depends on the internal structure of the test piece. As the rate of moisture absorption accelerated when the content exceeded 2% across all the test piece types, a 2% moisture content appears to represent a threshold value above which deterioration accelerated, at least in our test piece types formed under the IFP 80% condition. We propose that the deterioration mechanism proceeds in two distinct stages. In the first stage, water permeates from the surface and is absorbed, leading to material degradation and structural deterioration. In the second stage, decomposed material elutes from the structure and is replaced by the immersion solution, causing further deterioration.

A two-stage deterioration process was observed in the IFP 80% test pieces, but not in the IFP 100% test pieces, in this study. The major difference lay not in the outer shape of the structure, which remained consistent across the test piece types, but in the effective dimensions of the material elements that contacted moisture. In the parallel (P.) scan pattern, the load bearing section of the IFP 100% test piece functioned as a solid rod with a cross section of 3 × 3 mm (Section 2.1.). With IFP 80%, however, each structural element behaved more like a thin plate measuring 0.4 × 3 mm (Figure 11). Since PLA deterioration proceeded primarily through hydrolysis, the interaction between the polymer and moisture became critical, with the moisture penetration into the structure’s center governed by diffusion laws. This explains why the deterioration rates accelerated significantly in the IFP 80% structures. The observed two-stage deterioration manifested in three distinct periods that correlated with our moisture content measurements: an initial rapid deterioration period, a middle plateau period, and a final rapid deterioration period. We must account for the PLA crystallinity to fully explain this phenomenon, as the degree of crystallization influences the hydrolytic rate [46]. Further investigations are needed to develop a comprehensive model that can explain this unique deterioration pattern and provide a more complete understanding of the process.

### 4.4. Two-Stage Deterioration and Mitigation Strategies

Our results demonstrate that the deterioration proceeded in two stages and that the deterioration rate was higher at low IFP settings. The strength deteriorations resulting from these stages may pose challenges to the designer of a structure. To prevent first-stage deterioration, an outer shell can be constructed to shield against the immersion of water into the filaments within the structure. Such a shell can be constructed by adjusting the nozzle scan pattern in the FFF 3D printing software. From another perspective, internal gaps can be strategically designed to control the deterioration rate. For applications requiring rapid deterioration, internal gaps should be deliberately formed to facilitate water penetration. For structures requiring stability in immersion environments, the internal gaps should be minimized. These considerations may be important in the application 3D-printed PLA to tissue engineering.

On the topic of two-stage degradation, an earlier study reported that large lactide-glycolide implants degrade more slowly at their surface than in their interior [49]. Although the material compositions and test pieces differed in that study, their findings support our understanding of the degradation process.

### 4.5. Test Methods

The method used to test the mechanical deterioration inherently differs from study to study. Comparing the results among studies is difficult, as the experimental conditions seldom coincide.

In this study, we chose a PBS of pH 7.0 as the immersion solution for the test pieces. PBS retains its pH better than other immersion solutions, such as saline and ultrapure water. The pH of ultrapure water fluctuates easily in response to CO_2_ absorption during immersion, and the deterioration rate of a PLA structure varies correspondingly [39,50]. On the contrary, the deterioration rate of the structures immersed in PBS in our study did not largely differ from that previously reported in structures immersed in saline [37,38]. One earlier study assessed the influence of the medium type on the deterioration rate by comparing three degradation mediums, PBS, 0.9% NaCl, and distilled water [51]. They found that the type of medium influenced the deterioration of mechanical properties and the medium absorption during long-term degradation, and that PBS generally resulted in slower mechanical deterioration. Further investigation is needed to clarify how the immersion solution influences the deterioration.

We set the incubator temperature to 37 °C to facilitate comparison with previous data [37,38,51]. Other studies have set the immersion temperature at 21 °C and 70 °C to compare the deterioration rate and mechanism of decomposition [42], at 25, 30, 40, and 50 °C to evaluate the influence of temperature on PLA degradation [43], and at 60 °C [50].

The degradation observed in this study took place in two stages. As we only examined white PLA filament from a single supplier, we could not determine whether the same or similar results would have occurred with materials from other manufacturers. Previous research has documented how the PLA color affects the material properties of 3D-printed components [52]. Additional research will have to be conducted to resolve these questions.

One study focused on biomedical application proposed a standard protocol for the degradation of aliphatic poly-(α-hydroxy acids) in aqueous media to compare the mechanism by which PLA degrades [53].

In our tensile tests, we estimated Young’s moduli based on the slope of the stress–strain curve obtained from the displacement sensor in the testing apparatus. Though the accuracy of the strain estimate from a displacement sensor is clearly inferior to that from a strain gauge or extensometer, we could not apply these measurement devices to small-sized test pieces that had weakened through deterioration. We therefore surmise that the elastic moduli obtained in this paper were lower than the expected values [25,37,38]. Digital image correlation (DIC) analysis using an optical method could provide more precise estimates of the elastic modulus to determine the strain [54,55].

We modified the jig for the tensile test piece used in this study. The modified jig allowed testing of highly degraded test pieces with tensile strengths as low as 2.61 MPa after 120 days of immersion, representing only 5.07% of the strength of the non-immersed test piece (51.45 MPa for pattern P.) and a breaking load of 23.5 N. The tensile strength of test pieces formed by the IM method with ISO 527–1, type 1A geometry has been reported to deteriorate to 6.03 MPa from an initial value of 57.92 MPa (10.4% of the initial strength), corresponding to an estimated breaking load of 241.2 N [51]. The testing method using our newly developed jig enabled the tensile testing of test pieces that had decomposed to a breaking load of just 23.5 N, far below what was achievable in the previous report [51].

We estimated the moisture content using the loss-on-drying method in our study. While this method is inferior to Karl Fischer titration in precision, it is superior in convenience.

## 5. Conclusions

The deterioration of the mechanical properties of FFF-3D-printed structures with internal gaps was investigated by tensile testing after immersion in PBS for a maximum of 120 days. The rate at which the immersion degraded the strength was faster in structures with internal gaps than in fully filled structures. Variations in the nozzle scan pattern during fabrication changed the rate of water uptake into the structures. The water uptake and the strength decline proceeded in two stages, and the mechanical properties deteriorated correspondingly. The data obtained in this study will be useful for the design of PLA structures applied in tissue engineering.

## Data Availability

The original contributions presented in this study are included in the article. Further inquiries can be directed to the corresponding author.

## References

[1] Lunt J. (1998). Large-Scale Production, Properties and Commercial Applications of Polylactic Acid Polymers. Polym. Degrad. Stabil..

[2] Grillo A., Rusconi Y., D’Alterio M.C., De Rosa C., Talarico G., Poater A. (2024). Ring Opening Polymerization of Six- and Eight-Membered Racemic Cyclic Esters for Biodegradable Materials. Int. J. Mol. Sci..

[3] Singhvi M.S., Zinjarde S.S., Gokhale D.V. (2019). Polylactic acid: Synthesis and biomedical applications. J. Appl. Microbiol..

[4] DeStefano V., Khan S., Tabada A. (2020). Applications of PLA in modern medicine. Eng. Regen..

[5] Feng P., Jia J., Liu M., Peng S., Zhao Z., Shuai C. (2021). Degradation mechanisms and acceleration strategies of poly (lactic acid) scaffold for bone regeneration. Mater. Des..

[6] Yang Z., Yin G., Sun S., Xu P. (2024). Medical applications and prospects of polylactic acid materials. iScience.

[7] da Silva V.C., Gomes D.d.S., de Medeiros E.L.G., Santos A.M.d.C., de Lima I.L., Rosa T.P., Rocha F.S., de Souza Castro Filice L., Neves G.d.A., Menezes R.R. (2024). Highly Porous 3D Nanofibrous Scaffold of Polylactic Acid/Polyethylene Glycol/Calcium Phosphate for Bone Regeneration by a Two-Step Solution Blow Spinning (SBS) Facile Route. Polymers.

[8] Młotek M., Gadomska-Gajadhur A., Sobczak A., Kruk A., Perron M., Krawczyk K. (2021). Modification of PLA Scaffold Surface for Medical Applications. Appl. Sci..

[9] Zaszczyńska A., Moczulska-Heljak M., Gradys A., Sajkiewicz P. (2021). Advances in 3D Printing for Tissue Engineering. Materials.

[10] Rahatuzzaman M., Mahmud M., Rahman S., Hoque M.E. (2024). Design, fabrication, and characterization of 3D-printed ABS and PLA scaffolds potentially for tissue engineering. Results Eng..

[11] Zarea R.N., Doustkhahb E., Assadi M.H.N. (2021). Three-dimensional bone printing using hydroxyapatite-PLA composite. Mater. Today Proc..

[12] Hassanajili S., Karami-Pour A., Oryan A., Talaei-Khozani T. (2019). Preparation and characterization of PLA/PCL/HA composite scaffolds using indirect 3D printing for bone tissue engineering. Mater. Sci. Eng. C.

[13] Ismail R., Fitriyana D.F., Bayuseno A.P., Munanda R., Muhamadin R.C., Nugraha F.W., Rusiyanto, Setiyawan A., Bahatmaka A., Firmansyah H.N. (2023). Design, Manufacturing and Characterization of Biodegradable Bone Screw from PLA Prepared by Fused Deposition Modelling (FDM) 3D Printing Technique. J. Adv. Res. Fluid Mech. Therm. Sci..

[14] Raziyan M.S., Palevicius A., Perkowski D., Urbaite S., Janusas G. (2024). Development and Evaluation of 3D-Printed PLA/PHA/PHB/HA Composite Scaffolds for Enhanced Tissue-Engineering Applications. J. Compos. Sci..

[15] Salamanca E., Choy C.S., Aung L.M., Tsao T.-C., Wang P.-H., Lin W.-A., Wu Y.-F., Chang W.-J. (2023). 3D-Printed PLA Scaffold with Fibronectin Enhances In Vitro Osteogenesis. Polymers.

[16] Gasparotto M., Bellet P., Scapin G., Busetto R., Rampazzo C., Vitiello L., Shah D.I., Filippini F. (2022). 3D Printed Graphene-PLA Scaffolds Promote Cell Alignment and Differentiation. Int. J. Mol. Sci..

[17] Donate R., Paz R., Quintana Á., Bordón P., Monzón M. (2023). Calcium Carbonate Coating of 3D-Printed PLA Scaffolds Intended for Biomedical Applications. Polymers.

[18] Åkerlund E., Diez-Escudero A., Grzeszczak A., Persson C. (2022). The Effect of PCL Addition on 3D-Printable PLA/HA Composite Filaments for the Treatment of Bone Defects. Polymers.

[19] Balletti C., Ballarin M., Guerra F. (2017). 3D printing: State of the art and future perspectives. J. Cult. Herit..

[20] Pérez-Davila S., González-Rodríguez L., Lama R., López-Álvarez M., Oliveira A.L., Serra J., Novoa B., Figueras A., González P. (2022). 3D-Printed PLA Medical Devices: Physicochemical Changes and Biological Response after Sterilisation Treatments. Polymers.

[21] Grizzi I., Garreau H., Li S., Vert M. (1995). Hydrolytic degradation of devices based on poly(dl-lactic acid) size-dependence. Biomaterials.

[22] Vaid R., Yildirim E., Pasquinelli M.A., King M.W. (2021). Hydrolytic Degradation of Polylactic Acid Fibers as a Function of pH and Exposure Time. Molecules.

[23] Bogdanova A., Pavlova E., Polyanskaya A., Volkova M., Biryukova E., Filkov G., Trofimenko A., Durymanov M., Klinov D., Bagrov D. (2023). Acceleration of Electrospun PLA Degradation by Addition of Gelatin. Int. J. Mol. Sci..

[24] Song Y., Li Y., Song W., Yee K., Lee K.-Y., Tagarielli V.L. (2017). Measurements of the mechanical response of unidirectional 3D-printed PLA. Mater. Des..

[25] Tymrak B.M., Kreiger M., Pearce J.M. (2014). Mechanical Properties of Components Fabricated with Open-Source 3-D Printers Under Realistic Environmental Conditions. Mater. Des..

[26] Ambrus S., Soporan R.A., Kazamer N., Pascal D.T., Muntean R., Dume A.I., Mărginean G.M., Serban V.A. (2021). Characterization and mechanical properties of fused deposited PLA material. Mater. Today Proc..

[27] Chrysafi I., Ainali N.M., Bikiaris D.N. (2021). Thermal Degradation Mechanism and Decomposition Kinetic Studies of Poly(Lactic Acid) and Its Copolymers with Poly(Hexylene Succinate). Polymers.

[28] Chacón J.M., Caminero M.A., García-Plaza E., Núñez P.J. (2017). Additive manufacturing of PLA structures using fused deposition modelling: Effect of process parameters on mechanical properties and their optimal selection. Mater. Des..

[29] Hedayati R., Alavi M., Sadighi M. (2024). Effect of Degradation of Polylactic Acid (PLA) on Dynamic Mechanical Response of 3D Printed Lattice Structures. Materials.

[30] Koçar O., Anaç N., Baysal E., Parmaksız F., Akgül İ. (2024). Investigation of Mechanical Properties and Color Changes of 3D-Printed Parts with Different Infill Ratios and Colors After Aging. Materials.

[31] Travieso-Rodriguez J.A., Jerez-Mesa R., Llumà J., Traver-Ramos O., Gomez-Gras G., Rovira J.J.R. (2019). Mechanical Properties of 3D-Printing Polylactic Acid Parts subjected to Bending Stress and Fatigue Testing. Materials.

[32] Solomon I.J., Sevvel P., Gunasekaran J. (2021). A review on the various processing parameters in FDM. Mater. Today Proc..

[33] Öteyaka M.Ö., Aybar K., Öteyaka H.C. (2021). Effect of Infill Ratio on the Tensile and Flexural Properties of Unreinforced and Carbon Fiber-Reinforced Polylactic Acid Manufactured by Fused Deposition Modeling. J. Mater. Eng. Perform..

[34] Monaldo E., Ricci M., Marfia S. (2023). Mechanical properties of 3D printed polylactic acid elements: Experimental and numerical insights. Mech. Mater..

[35] Gunasekaran K.N., Aravinth V., Kumaran C.B.M., Madhankumar K., Kumar S.P. (2021). Investigation of mechanical properties of PLA printed materials under varying infill density. Mater. Today Proc..

[36] Zhang X., Chen L., Mulholland T., Osswald T.A. (2019). Characterization of mechanical properties and fracture mode of PLA and copper/PLA composite part manufactured by fused deposition modeling. SN Appl. Sci..

[37] Suzuki M., Yonezawa A., Takeda K., Yamada A. (2019). Evaluation of the Deterioration of the Mechanical Properties of Poly(lactic acid) Structures Fabricated by a Fused Filament Fabrication 3D Printer. Inventions.

[38] Yonezawa A., Yamada A. (2021). Deterioration of the Mechanical Properties of FFF 3D-Printed PLA Structures. Inventions.

[39] Farah S., Anderson D.G., Langer R. (2016). Physical and mechanical properties of PLA, and their functions in widespread applications—A comprehensive review. Adv. Drug Deliv. Rev..

[40] Ng F., Nicoulin V., Peloso C., Curia S., Richard J., Lopez-Noriega A. (2023). In Vitro and In Vivo Hydrolytic Degradation Behaviors of a Drug-Delivery System Based on the Blend of PEG and PLA Copolymers. ACS Appl. Mater. Interfaces.

[41] von Burkersroda F., Schedl L., Göpferich A. (2002). Why degradable polymers undergo surface erosion or bulk erosion. Biomaterials.

[42] Banjo A.D., Agrawal V., Auad M.L., Celestine A.-D.N. (2022). Moisture-induced changes in the mechanical behavior of 3D printed polymers. Compos. Part C Open Access.

[43] Deroiné M., Duigou A.L., Corre Y.-M., Gac P.-Y.L., Davies P., César G., Bruzaud S. (2014). Accelerated ageing of polylactide in aqueous environments: Comparative study between distilled water and seawater. Polym. Degrad. Stab..

[44] Oliver-Ortega H., Tarrés Q., Mutjé P., Delgado-Aguilar M., Méndez J.A., Espinach F.X. (2020). Impact Strength and Water Uptake Behavior of Bleached Kraft Softwood-Reinforced PLA Composites as Alternative to PP-Based Materials. Polymers.

[45] Moliner C., Finocchio E., Arato E., Ramis G., Lagazzo A. (2020). Influence of the Degradation Medium on Water Uptake, Morphology, and Chemical Structure of Poly(Lactic Acid)-Sisal Bio-Composites. Materials.

[46] Klimczuk B., Rudnicka A., Owczarek O., Puszkarz A.K., Szparaga G., Puchalski M. (2024). Investigation of the Hydrolytic Degradation Kinetics of 3D-Printed PLA Structures under a Thermally Accelerated Regime. Materials.

[47] Matsusue Y., Yamamuro T., Oka M., Shikinami Y., Hyon S.-H., Ikada Y. (1992). In vitro and in vivo studies on bioabsorbable ultra-high-strength poly(L-lactide) rods. J. Biomed. Mater. Res..

[48] Makino K., Arakawa M., Kondo T. (1985). Preparation and in Vitro Degradation Properties of Polylactide *Microcapsules*. Chem. Pharm. Bull..

[49] Therin M., Christel P., Li S., Garreau H., Vert M. (1992). In vivo degradation of massive poly(α-hydroxy acids): Validation of In vitro findings. Biomaterials.

[50] Li S., McCarthy S. (1999). Further investigations on the hydrolytic degradation of poly (DL-lactide). Biomaterials.

[51] Andrzejewska A. (2019). One Year Evaluation of Material Properties Changes of Polylactide Parts in Various Hydrolytic Degradation Conditions. Polymers.

[52] Wittbrodt B., Pearce J.M. (2015). The effects of PLA color on material properties of 3-D printed components. Addit. Manuf..

[53] Li S.M., Garreau H., Vert M. (1990). Structure-property relationships in the case of the degradation of massive aliphatic poly-(α-hydroxy acids) in aqueous media, Part 1: Poly(dl-lactic acid). J. Mater. Sci. Mater. Med..

[54] Sutton M.A., Orteu J.-J., Schreier H. (2009). Image Correlation for Shape, Motion and Deformation Measurements. Basic Concepts, Theory and Applications.

[55] Sutton M.A., Wolters W.J., Peters W.H., Ranson W.F., McNeill S.R. (1983). Determination of displacements using an improved digital correlation method. Image Vis. Comput..

